# Clinicopathologic review of polyps biopsied at colonoscopy in Lagos, Nigeria

**DOI:** 10.11604/pamj.2016.24.333.9434

**Published:** 2016-08-30

**Authors:** Aderemi Oluyemi, Nicholas Awolola, Olufemi Oyedeji

**Affiliations:** 1 ReMay Consultancy & Medical Services, Ikeja, Lagos, Nigeria; 2 College of Medicine, University of Lagos, Idi-Araba, Lagos, Nigeria

**Keywords:** Polyps, colonoscopy, adenoma, Nigeria

## Abstract

**Introduction:**

Colorectal polyps are known precursors of colorectal cancers. The increase in utilization of colonoscopy in Nigeria has meant a rise in the recently reported incidence of these lesions. The aim of this study is to evaluate the clinicopathological profile of colorectal polyps biopsied during the inaugural 12 month period of colonoscopy from a private endoscopy suite in Nigeria.

**Methods:**

This is a retrospective review of all the clients who had polyps diagnosed at colonoscopy over a 12 month period (August 2014 –July 2015) at a private endoscopy suite in Lagos, Nigeria. This analysis of prospectively collected data was performed using clinical information from the endoscopy logs and pathology database system of a private endoscopy suite based in Lagos, Nigeria.

**Results:**

A total of 125 colonoscopies were carried out over the stated period. Of these, 14 individuals had a total of 18 polyps- 4 clients (28.6% of the persons with polyps) had two polyps each. The polyp detection rate was 11.2% while the polyp per colonoscopy rate was 14.4%. Of these clients, males were 10 in number; giving a male to female ratio of 2.5:1. Their ages ranged from 37 to 77 years (mean= 57.3 years). The presenting complaint at colonoscopy was hematochezia in 11 (78.6%), new onset constipation in 2 (14.2%) and peri-anal pain in 1 patient (7.1%). The polyps were distributed as follows; 2 (11.1%) in the ascending colon, 1 (5.6%) each in the transverse and descending colons, 8 (44.4%) in the sigmoid colon, 6(33.3%) located in the rectum. Hence, there was left sided (15 of 18= 83.3%) preponderance. Pathologically, tubular (adenomatous) polyp with or without low grade dysplastic changes was diagnosed in 6 of the 18 polyps (giving an adenoma detection rate of 4.8%), 4 (22.2%) were inflammatory polyps, 1 (5.6%) was malignant and another had the rare inflammatory fibroid polyp. Five (27.8%) of the specimens were reported as non-specific colitis.

**Conclusion:**

The study supports the present wisdom that polyps are clearly less prevalent in our environment when compared to the Western world. The increased prevalence with advancing age, in male subjects and of left sided lesions, is also in keeping with previous results from our environment. A case is also advanced for the increased deployment of endoscopy as a tool for the detection of these polyps and ultimately, the reduction of colorectal cancer in our population.

## Introduction

The burden of colorectal cancer (CRC) is huge in terms of morbidity and mortality throughout the world. They account for over 9% of all cancer incidence, are the third most common cancers worldwide and the fourth most common cause of cancer-related deaths [1, 2]. A majority of CRC are thought to arise through a multi-step pathway which includes initial hyper-proliferation of normal epithelial cells, formation of adenomas and finally the transition to invasive carcinomas [3, 4]. Detection of these polyps at colonoscopy and polypectomy has been shown to significantly reduce the incidence of CRC- by interrupting this adenoma-carcinoma sequence [5]. This is what gives particular relevance to the detection of polyps at colonoscopy. There has been little by the way of locally-based publications that have specifically focused on this important entity. However, a welcome development has been the recent rise in data concerning the prevalence of polyps in this region of the world- both as directly designed studies and as part of various colonoscopy audits [6–12]. This study was aimed at determining the prevalence of polyps among patients who underwent a colonoscopy at a private center in Lagos, Nigeria and to discuss their clinical features and the pathological findings.

## Methods

**Study design**: this is a retrospective, hospital-based review.

**Study methods**: This retrospective analysis of prospectively collected data was performed using clinical information from the endoscopy logs and pathology database system of a private endoscopy suite in Lagos, Nigeria. All consecutive patients who had polyps diagnosed at colonoscopy over a 12 month period (August 2014 –July 2015) were included.

During the procedure, the colonic polyps were characterized by the following: location----the anatomical site of the polyp was documented; number---- whether the polyps were single or multiple was noted; appearance--- the masses were classified as either sessile or pedunculated.

**Data presentation and definition of terms**: The data is presented as simple proportions and the terms used are defined here as: Polyp Detection Rate- this is the number of colonoscopies in which polyps were detected taken as a proportion of the total number of such procedures that were carried out; Polyps per Colonoscopy Rate- this is the total number of polyps detected at colonoscopy taken as a proportion of the total number of such procedures that were carried out; Adenoma Detection Rate- this is the number of polyps that were histologically diagnosed as adenomatous taken as a proportion of the total number of colonoscopies.

## Results

**Figure 1:**
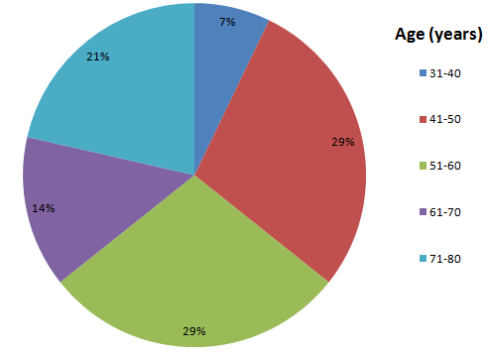
Pie chart showing age distribution of patients

**Figure 2:**
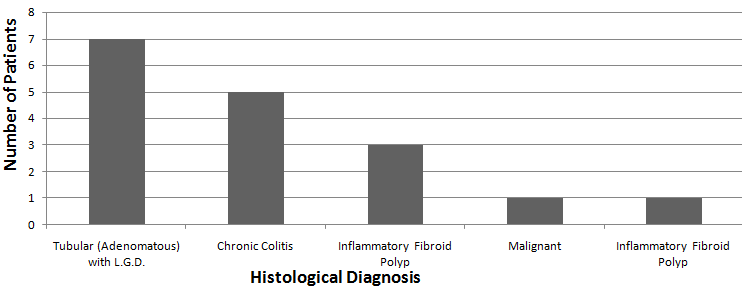
Bar chart showing the histological diagnosis of polyps, (LGD= low grade dysplasia)

One hundred and twenty five colonoscopies were performed over the 12 months covered by the study. There were 100 males; giving a male to female ratio of 2.5:1. Their ages ranged from 37 to 77 years (mean= 57.3 years) with majority of the patients within the 41-60 years age range (Figure 1). There was no statistically significant difference in the ages of both sexes. Fourteen individuals (11.2%) had a total of 18 polyps- 4 clients (28.6% of the people with polyps) had 2 polyps each. The major reason for colonoscopy referral was hematochezia as seen in 10 of the patients, the others were change in bowel habit in 3 patients while one was referred on account of perianal pain (Table 1). The polyps were distributed as follows in the colon; 8 in the sigmoid, 6 in the rectum, 1 each in the descending and transverse colon, and 2 in the ascending colon (Table 2). Hence, there was left sided (15 of 18= 83.3%) preponderance. Additional findings at colonoscopy included hemorrhoids 5(25.7%) patients in and diverticulosis in 2(14%). Two of the 18 polyps (11.1%) were semi-sessile and polypoidal while the rest were sessile (77.8%). Figure 2 illustrates the pattern of histological diagnoses that were made. Eight of the 18 polyps (44.4%) were tubular (adenomatous) with low grade dysplasia in all of them. Chronic colitis and inflammatory polyp were the reports in 5(27.8%) and 4(22.2%) patients respectively while 1(5.6%) each was reported as malignant and the rare inflammatory fibroid polyp. Thus the adenoma detection rate (ADR) was 6.4%. All the adenomatous lesions were located in the rectosigmoid.

**Table 1:** Showing the reasons for colonoscopy referral

Reason for Colonoscopy	Number of Patients
Hematochezia	10
Change in Bowel Habits	3
Perianal Pain	1

**Table 2:** Showing colonic distribution of polyps

Location in Colon	Number of Polyps (n=18)
Ascending	2
Transverse	1
Descending	1
Sigmoid	8
Rectum	6

## Discussion

There are no published population-based data on the prevalence of polyps in our setting, the range of quoted figures vary from very rare (e.g. 1 % from Okitipupa in South-West, Nigeria) to much more prevalent (e.g. 16.2% from Ile-Ife in South-West, Nigeria) but all are from hospital-based studies. The findings from some previous studies from our region are summarized in Table 3. A PDR of 11.2% is reported from this study. This figure is much higher than some earlier studies and lower than some recent ones [6–12]. The contrasting figures of PDR is likely due, at least in some part, to the variations in location and therefore predominant ethnic groups and the wide range in population sample sizes. All of the patients with polyps were symptomatic at the time of colonoscopy. This is in keeping with findings from local and international studies [6, 13]. A recent large, prospective, multicentre study concluded that the rates of detected polyps and advanced adenomas were much higher in symptomatic individuals [13]. Another important result of this study was that there was no significant difference in the CRC rates in asymptomatic patient’s vrs symptomatic ones. Hence, the researchers concluded that these results reinforce the importance of screening the symptom-free population to prevent CRC. Histologically, colorectal polyps are classified mainly as adenoma (neoplastic) or hyperplastic (non-neoplastic) [14]. Alatise and his team are the only ones to have previously documented ADR at colonoscopy in Nigerians from their study of polyps in Ile-Ife [6]. Their figure of 6.8% from 415 colonoscopies is similar to that presented in this study-6.4%, but much higher than the 2.9% quoted from Ghana by Dakubo et al [12]. This data is quite low when compared with larger surveys of Americans and Asians [15, 16]. Perhaps this data reflects the difference in prevalence rates of CRC across the continents- as the adenoma-carcinoma sequence is still thought to be a major etiopathogenetic mechanism for the development of CRC. The significance of these low ADR figures from our locality in terms of its long term correlation with the development of CRC is still under debate. The enquirers ask why such low ADR figures should explain the perceived increase in the number of CRC cases [6]. The adenoma-carcinoma sequence as the explanation for CRC’s etiopathogenesis in this population has been questioned and alternative route or routes to the cancer’s development opined [17–19]. The rectosigmoid was the site in 77% of the polyps, and all the adenomatous lesions were also in this part of the colon. This result parallels the observed patterns of anatomic distribution of CRC from various loco-regional reports [12, 20–22]. The findings would therefore suggest that the use of rectosigmoidoscopy as an endoscopy tool for CRC screening in our locality is most welcome. Adenomatous polyps are still widely regarded as premalignant and rectosigmoidoscopy in this series would have picked 100% of them. The procedure has consistently been shown in prospective studies to be most useful in the fight against morbidity and mortality of CRC. A range of studies from across the globe have shown improved outcomes in patients who had as little as a single rectosigmoidoscopy done before the age of 50 and this benefit is said to increase with another such screen years after [23–29]. Bearing in mind that a recent mathematical modelling study showed that in the sub-Saharan African region, screening for CRC by colonoscopy at age 50 years in combination with treatment can be considered cost-effective and that the present rates of CRC screening of any kind in our environment are abysmal [30, 31], rectosigmoidoscopy would appear to be a most screening tool. Particularly as it has the added advantage that it is cheaper, faster, easier to perform, training requirements are less and the rigors of bowel preparation demands are much reduced. So consistent are the results from other environments quoted earlier that only one of the referenced studies showed no benefit for rectosigmoidoscopy after long term follow up [27]. However, further analysis of the negative study did show that mortality from CRC was lower in the sub-population of hospital attenders- a reduction of 59% for any location of CRC and 76% for rectosigmoidal cancer [27].

**Table 3:** Showing findings from similar loco-regional studies

Article Author (Date)	Study Site	Total Number of Patients (n)	Polyp Detection Rate (%)
**Alatise et al** **(2014)**	Ile-Ife, South-West, Nigeria	415	16.2
**Ajayi et al^&^** **(2014)**	Ado-Ekiti, South-West, Nigeria	68	14.7
**Onyekwere et al** **(2013)**	Lagos, South-West, Nigeria	607	6.8
**Ismaila et al** **(2013)**	Jos, North Central, Nigeria	68	10.2
**Olokoba et al** **(2013)**	Ilorin, North-Central, Nigeria	103	15.5
**Obonna et al** **(2012)**	Okitipupa, South-West, Nigeria	100	1
**Dakubo et al** **(2008)**	Accra, Ghana	586	2.9*

* Adenomatous polyps alone were noted

& The population consisted primarily of patients with lower gastrointestinal bleeding

## Conclusion

The study supports the present wisdom that polyps are clearly less prevalent in our environment when compared to the Western world. The preponderance of males, advancing age and left sided lesions is also in keeping with previous results from our environment. The institution of cost-effective population based CRC screening programmes in our locality is espoused along with a suggestion of the deployment of endoscopy as a useful tool for such programmes.

### What is known about this topic

- Colorectal polyps are precursors to cancers;
- Screening for these cancers are virtually none existent in the better part of the African continent.

### What this study adds

- Presents fresh data from Nigeria about colorectal polyp characterization;
- Examines the possible benefits of introduction of such screening programmes.
